# Functional Coupling within the Mesolimbic Circuit in First-Episode Psychosis

**DOI:** 10.1523/ENEURO.0097-21.2021

**Published:** 2021-04-21

**Authors:** Or Duek

**Affiliations:** Departement of Psychiatry, Yale University School of Medicine, New Haven, CT, 06510

Psychosis disorder is a debilitating disorder characterized by multiple admissions to psychiatric care facilities, higher unemployment rates, and decreased life expectancy. All of which create a high burden on the patient, their family, and the healthcare system. Understanding the basic mechanisms of psychotic disorder is essential for early discovery and facilitating better care for those suffering from it (Correll et al., 2018).

The dopamine hypothesis of schizophrenia (Howes et al., 2009) suggests that dysregulation in dopamine signaling is the main cause of psychosis. Consistent with this theory, the ventral-tegmental area (VTA) is a pivoting region in psychosis in both animals and humans (Modinos et al., 2015). Individuals suffering from psychosis have elevated dopaminergic activity in the VTA. Such dysregulations were also found in prodromal syndromes (before full-blown development of psychosis; Howes et al., 2009), suggesting a causal effect of dopaminergic dysregulation in psychosis. In animal models of psychosis, hyperactivity of the hippocampus was associated with upregulation of tonic activity in the VTA (Modinos et al., 2015), suggesting that the dopaminergic dysregulation might be presented within the coupling of the hippocampus and VTA, not only their activation. One aspect of the aberrant functional connectivity in the mesolimbic circuit that was less studied is during first-episode psychosis. Such examination might shed light on possible neural mechanisms that underlie psychosis, especially as in first-episode psychosis, people are less likely to be influenced by medications or the chronicity of the disease.

A recent *eNeuro* paper by Gregory et al. (2021) was set to do exactly this. The authors examined the functional connectivity in the mesolimbic circuit at resting state, in individuals suffering from first-episode psychosis and healthy controls. Their analysis focused on the three main regions of interest in that circuit: VTA, hippocampus, and nucleus accumbens (NAc). This analysis revealed higher functional connectivity between VTA and right anterior hippocampus in individuals with first-episode psychosis; the connectivity strength was also correlated with psychotic symptoms, measured by the Brief Psychiatric Rating Scale. Conversely, functional connectivity between VTA and NAc was reduced in individuals with first-episode psychosis compared with controls.

Increased functional connectivity of VTA with the hippocampus and decreased functional connectivity of the same brain structure with the striatum were also found in individuals at high risk for psychosis in a task-based paradigm (Modinos et al., 2020). In the task, participants looked at novel versus neutral stimuli. Combining the findings from Modinos et al. (2020) and the recent *eNeuro* paper by Gregory et al. (2021) can help us understand the paradoxical patterns of psychosis disorder. Some of the symptoms are associated with the upregulation of dopamine (delusions and hallucinations), while others are associated with downregulation (negative symptoms such as anhedonia). The higher VTA-hippocampus coupling and lower VTA-striatal coupling in individuals who either suffer from psychosis or are at risk for the disorder, compared with healthy controls, suggest a possible neural mechanism for this paradox.

Studies in individuals at later stages of psychotic disorders show some differences in the pattern of results. A recent study by Nakamura et al. (2020) did not find a difference in VTA-hippocampus functional connectivity between individuals diagnosed with schizophrenia and healthy controls, at resting state. They did find a positive correlation between this coupling and positive symptoms, in the individuals suffering from psychosis. Taken together, these differences may point to the effect of chronicity, medication, or other variables in the course of psychosis illness, all of which emphasize the relevance of examination in the early stages of psychosis.

This recent paper presents a promising and interesting direction in understanding the mechanisms associated with psychosis, by evaluating resting-state functional connectivity in first-episode psychosis. The alignment of these resting-state findings with task-based results could point toward a possible direction for further investigation. Future studies may focus on longitudinal data, using the previous findings as a model to predict the onset, risk, and course of psychosis based on both task-based and resting-state connectivity. Such a model will enable us to promote better care for individuals suffering from psychosis and their families.

## Synthesis

Reviewing Editor: Christophe Bernard, INSERM & Institut de Neurosciences des Systèmes
